# Virulence characteristics of *hcp*^+^*Campylobacter jejuni* and *Campylobacter coli* isolates from retail chicken

**DOI:** 10.1186/s13099-015-0067-z

**Published:** 2015-07-24

**Authors:** Nicolae Corcionivoschi, Ozan Gundogdu, Lynn Moran, Carmel Kelly, Pam Scates, Lavinia Stef, Ada Cean, Brendan Wren, Nick Dorrell, Robert H Madden

**Affiliations:** Agri-Food and Biosciences Institute, Food Microbiology, Newforge Lane, Belfast, BT9 5PX UK; School of Animal Science and Biotechnology, Banat University of Animal Sciences and Veterinary Medicine-King Michael I of Romania, Calea Aradului nr. 119, Timisoara, Romania; London School of Hygiene and Tropical Medicine, London, UK

**Keywords:** *Campylobacter jejuni*, Retail chicken, Type VI secretion system

## Abstract

**Background:**

Recently the Type VI secretion system (T6SS), which can play a significant role in bacterial survival and pathogenesis, was reported in *Campylobacter* spp., having the *hcp* gene as a key component.

**Methods:**

Campylobacteriosis is associated with the consumption of infected chicken meat. Our study aimed to explore the presence of T6SS in *C. jejuni* (n = 59) and *C. coli* (n = 57) isolates, from retail raw chicken and to investigate their pathogenic potential. The *hcp* gene was used as an indicator for the T6SS presence.

**Results:**

Using multiplex PCR we have identified a significantly higher prevalence of *hcp* in *C. coli* isolates (56.1%) than in *C. jejuni* (28.8%) and AFLP analysis of the isolates showed a high degree of genetic similarity between the isolates carrying the *hcp* gene. Genome sequencing data showed that 84.3% of the *C. coli* and 93.7% of the *C. jejuni* isolates had all 13 T6SS open reading frames. Moreover, the virulence characteristics of *hcp* + isolates, including motility and the ability to invade human intestinal epithelial cells in vitro, were significantly greater than in the control strain *C. jejuni* 12502; a human isolate which is *hcp* positive.

**Conclusion:**

Overall, it was discovered that *hcp*^+^*C. coli* and *C. jejuni* isolated from retail chicken isolates posses genetic and phenotypic properties associated with enhanced virulence. However, since human infections with *C. coli* are significantly less frequent than those of *C. jejuni*, the relationship between virulence factors and pathogenesis requires further study.

## Background

*Campylobacter* species are the most common foodborne pathogens in humans but also commensals in many farm animals, including cattle, swine and poultry. *Campylobacters* are Gram-negative, microaerophilic microorganisms possessing a corkscrew motility phenotype which has proven significance in achieving penetration of the human gut epithelium in order to establish infection in humans, and colonisation in poultry [1, 2].

The most frequent cause of campylobacteriosis in the UK is poultry meat [3] and up to 90% of poultry carcasses are contaminated with *C. jejuni* [4, 5] and *C. coli* [6]. The virulence properties of campylobacters are, however, yet to be fully defined and will be dependent on the genetic content of each individual isolate. *Campylobacters* can cause a range of illnesses; diarrhoea, reactive arthritis and in some cases infection can subsequently lead to serious neuromuscular disorders such as Guillain-Barré syndrome [7, 8]. One pathogenic property employed by bacteria and reported in *Campylobacter* spp., is the use of secretion systems to export toxins (proteins) into their environment, or directly through membranes into neighbouring eukaryotic [9] or prokaryotic [10] cells.

The Type VI secretion system (T6SS), has been reported in *Campylobacter* [11], as well as other Gram negative bacteria [12]. In *C. jejuni* this secretion system is potentially associated with more severe forms of disease as it can confer cytotoxicity toward red blood cells [11]. A significant component of the T6SS is the product of the *hcp* gene and the presence of this gene has been described as being indicative of a functional T6SS in *C. jejuni* [13]. Further, it has been reported as being more prevalent in strains isolated from patients experiencing bloody diarrhoea, than those having non-bloody diarrhoea [13]. Also, *hcp*^+^ strains of *C. jejuni* have shown increased abilities to adhere to, and invade, the host gastrointestinal epithelium in vivo [14].

This study has focused only on the *hcp*^+^ isolates as information on their virulence is lacking for this emerging virulence mechanism. This study aimed to analyze the *C. jejuni* and *C. coli* isolates obtained from retail packs of raw chicken produced in Northern Ireland for the presence of the T6SS and to test their pathogenic potential.

## Methods

### Microbiology

All media were supplied by Thermo Fisher Scientific Ltd, Basingstoke UK, and all reagents supplied by Sigma-Aldrich Ltd, Gillingham, UK unless otherwise stated. All incubations were performed microaerobically (85% N_2_, 10% CO_2_ and 5% O_2_, all v/v) in a Don Whitley MACS 500 workstation (Don Whitley Scientific, Shipley, UK) at 41.5°C unless otherwise stated.

### *Campylobacter* isolation and identification

Samples of retail chicken were prepared as previously described [15]. Briefly, we have used a stomacher bag and buffer peptone water (225 ml) to emulsify 25 g of skin and flesh sample in a Seward 400 blender (Seward Ltd, Worthing, UK). The emulsified sample (25 ml) was transferred into a container with 225 ml of BBW (Bolton broth). The BS EN ISO10272-1:2006 [16] was followed as previously described [17]. The 225 ml BB were first incubated for 4 h at 37°C, followed by a second incubation step of 24 h at 41.5°C. The resulting culture was plated on modified charcoal cefoperazone deoxycholate agar (mCCDA) and incubated at 41.5°C until single colonies were countable. In order to confirm that the resulting colonies represent a typical *Campylobacter* colony the motility and oxidase tests were performed. DNA was extracted from each individual isolate using half of a 10 μl loopful in 1 ml of SET buffer (150 mmol l^−1^ NaCl, 15 mmol l^−1^ EDTA, 10 mmol l^−1^ Tris–HCl, pH 8.0). Long-term stocks (−80°C) were prepared in 1 ml of NB plus (nutrient broth plus) containing 10% (v/v) glycerol Overall 59 *C. jejuni* and 57 *C. coli* poultry isolates from Northern Ireland producers were selected from the culture collection yielded by the survey [15] and investigated in this study.

### Genome sequencing, assembly and annotation

We have performed genome sequencing of all our 52 [18] *C. jejuni* and *C. coli* isolates as described previously by Ugarte Ruiz et al. [19] using Illumina MiSeq 2 × 150 bp paired-end sequencing. To analyze the data quality FastQC was used [20]. In order to evaluate the sequencing reads the Trimmomatic was used at the following parameters: (v0.32) ‘leading’ and ‘trailing’ setting of 3, a ‘slidingwindow’ setting of 4:20 and a ‘minlength’ of 36 nucleotides) [21]. BWA-MEM (v0.7.7-r441) was used to map the reads using the genome sequence of *C. jejuni* NCTC 11168 (AL111168) as Ref. [22]. Velvet Optimiser (v2.2.5) using n50 optimization [23, 24] was used to perform assembly. The reference strain *C. jejuni* NCTC 11168 (AL111168) was used to complete Contigs using ABACAS (v1.3.1) [25]. In order to finalize annotation of all the genomes we have used RATT [26] and the references species *C. jejuni* NCTC 11168 (AL111168), *C. jejuni* 414 (CM000855), *C. jejuni* RM1221 (CP000025), *C. coli* 76339 (HG326877), *C. coli* CVM N29710 (CP004066), *C. concisus* 13826 (CP000792), *C. fetus* 82-40 (CP000487), *C. jejuni* 81-176 (CP000538), *C. jejuni* M1 (CP001900) and *C. lari* RM2100 (CP000932). The Artemis and ACT software [27] were used to read the genomes. T6SS ORFs were identified using BLAST [28, 29].

### Amplified fragment length polymorphism (AFLP)

The ABI 3100 Genetic Analyzer was used to perform automated amplified fragment length polymorphism (AFLP) as previously described [30, 31]. The resulting data was analysed using the BioNumerics V4.6.1 software and the Pearson product-moment correlation coefficient was required to normalize the profile similarities. Following normalization the unweighted pair group with mathematical average (UPGMA) method was used for clustering and dendrogram assembly. As controls DNA from standard *C. jejuni* and *C. coli* cultures were used in each run. In our experiments the similarities between standards were ≥90% having a position tolerance setting of 0.07%, and a profile size range of 50–500 bp.

### PCR detection of *hcp* and *gltA* genes

To detect the *hcp* multiplex PCR analysis was used as previously described using the *gltA* as a control housekeeping gene [13]. For amplification of *gltA* the primers gltAF (gcccaaagcccatcaagcgga) and gltAR (gcgctttggggtcatgcaca) and for the amplification of the *hcp* gene primers hcpF (caagcggtgcatctactgaa) and hcpR (taagctttgccctctctcca) were used. *C. jejuni* NCTC 12502 served as the hcp^+^ control.

### Motility assay

The motility of all *hcp*^+^ isolates (n = 51), plus one *hcp*^−^ strain and NCTC 12502, was compared based on the method of Corcionivoschi et al. [32]. Briefly, 5 µl of culture [48 h, grown on blood agar and recovered in 1 ml brain heart infusion (BHI) broth] was inoculated into the centre of semi-solid BHI plates (0.4% agar). The radius of the disc of visible growth was measured after incubation (48 h). The experiment was carried out in triplicate.

### Resistance to bile salts

The resistance of the isolates to bile salts was studied based on the method of Stef et al. [33]. Briefly, each isolate was grown confluently on blood agar (48 h) then 2.5 ml BHI broth added and mixed. This cell suspension (1 ml) was used to inoculate BHI broth (10 ml), and BHI broth containing 4.0% (wt/vol) bile salts (10 ml), dispensed into 30 ml bottles. The bottles were incubated (24 h) after which 20 μl was removed and added to 100 μl of water. Serial dilutions were made in maximum recovery diluent, plated onto mCCDA, incubated (48 h), and enumerated. The experiment was carried out in triplicate. Due to complete lack of resistance of some of the isolates we have scored the resistance as (+) resistant, (±) intermediate and (−) sensitive.

### Gentamicin protection assay

In order to quantify the invasive capability of *C. jejuni* and *C. coli* isolates the gentamicin protection assay has been used as previously described [34]. Intestinal epithelial cells (HCT-8) were grown for up to 18 h until a confluence of 1 × 10^5^ has been reached. The bacterial isolates were cultivated on blood agar plates for 2 days followed by serial dilution to reach an OD_600_ of 0.4. Once the HCT-8 cells reached the desired confluence they were washed with 1 ml of PBS followed by the addition and 2 ml of fresh tissue culture media. In our current study an MOI (multiplicity of infection) of 10 was used. One centrifugation step was necessary (250×*g* for 5 min) followed by incubation for 3 h at 37°C, in microaerophilic conditions (10% CO_2_). The infected cells, previously treated with 400 μg/ml, were washed three times in 1 ml PBS followed by exposure to 0.1% Triton X-100 at 37°C during a 2 h period. The lysate was diluted and spread on Mueller–Hinton agar plates and colonies counted after 2–3 days incubation or until colonies were visible on plates. The ratio between the total number of CFU and the initial inoculum was used to calculate the efficiency of invasion. The experiments were performed in triplicate.

### Visualisation of capsular polysaccharide (CPS)

CPS was extracted from the isolates using the method of Hitchcock [35]. Briefly, plate grown bacteria were lysed for 5 min at 100°C in 31.25 mM Tris–HCl (pH 6.8), 4% sodium dodecyl sulphate, 0.025% bromophenol blue, and 20% glycerol. Proteinase K (20 mg ml^−1^ proteinase) was added to the lysate and incubated for 1 h at 50°C. The lysate, containing the polysaccharides, were separated in 10% Bis–Tris gels (NuPage Novex) for 1 h at 100 V (Invitrogen, Paisley, United Kingdom) and he gels were stained with Alcian blue [36].

### Statistical analysis

Experiments were repeated three times in separate instances. Means of standard deviations (±) were used to represent the repeated experiments. Prism software was required for graph design and the unpaired Student *t* test to calculate the significance of data. The resulting P values were considered significant if their calculated values were <0.05.

## Results

### Prevalence of *hcp* and identification of T6SS open reading frames in *C. coli* and *C. jejuni* isolates

**Figure 1:**
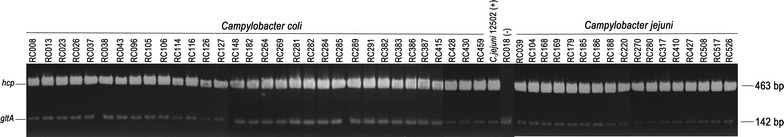
PCR detection of *hcp* in *C. jejuni* (n = 17) and *C. coli* (n = 32) isolates. Only positive isolates are shown, and one negative isolate (RC018). The positive control for *hcp* was *C. jejuni* 12502 and *gltA* served as the negative control for PCR reaction. Photoshop software was used to achieve the desired resolution.

Using PCR, the presence of the *hcp* gene in the *C. jejuni* and *C. coli* isolates obtained packed retail chicken produced in Northern Ireland was determined, Figure 1. A higher prevalence was found in *C. coli* isolates (56.1%) than in *C. jejuni* (28.8%), Table 1. The *gltA* housekeeping gene in the multiplex PCR served as a positive control, and confirmed the isolates were all *Campylobacter* spp. *C. jejuni* strain 108 (JX436460) was used as reference when analysing the genomes of the chicken isolates in order to identify the presence of the T6SS ORFs. The *hcp*^+^ isolates were next investigated for the T6SS ORFs integrity. The results showed that 84.3% of the *C. coli* and 93.7% of the *C. jejuni* isolates, detected positive for *hcp*, possess all 13 T6SS ORFs, Table 2.

**Table 1:** Prevalence of hcp gene in *Campylobacter* spp. isolated from raw, retail chicken

Species	Total number of isolates	Number of *hcp* positive strains (%)
*Campylobacter coli*	57	32 (56.1)
*Campylobacter jejuni*	59	17 (28.8)

**Table 2:** Presence of T6SS components in *C. coli* and *C. jejuni* chicken isolates in comparison with the amino acid sequence matches of *C. jejuni* strain 108

	TssA	TssB	TssC.	TssD	TssE	TssF	TssG	TssH	TssI	TssJ	TssK	TssL	TssM		
Amino acids	415	161	484	171	130	573	302	299	838	148	465	257	1175		
REFERENCE	TssA	TssB	TssC	TssD	TssE	TssF	TssG	TssH	TssI	TssJ	TssK	TssL	TssM	PCR results	Spp.
RC008														+	C.colli
RC013														+	C.colli
RC023	412 (99.3%)	160 (99.4%)	481 (99.4%)	171 (100%)	126 (97.0%)	568 (99.1%)	302 (100%)	294 (98.3%)	758 (90.5%)	147 (99.3%)	464 (99.8%)	257 (100%)	1171 (99.7%)	+	C.colli
RC026														+	C.colli
RC037	412 (99.3%)	160 (99.4%)	481 (99.4%)	171 (100%)	126 (97.0%)	567 (99.0%)	302 (100%)	294 (98.3%)	821 (98.0%)	147 (99.3%)	464 (99.8%)	257 (100%)	1170 (99.6%)	+	C.colli
RC043	412 (99.3%)	160 (99.4%)	481 (99.4%)	171 (100%)	126 (97.0%)	567 (99.0%)	302 (100%)	294 (98.3%)	821 (98.0%)	147 (99.3%)	464 (99.8%)	257 (100%)	1170 (99.6%)	+	C.colli
RC096	412 (99.3%)	160 (99.4%)	481 (99.4%)	171 (100%)	126 (97.0%)	567 (99.0%)	302 (100%)	294 (98.3%)	791 (94.4%)	147 (99.3%)	436 (93.8%)	257 (100%)	1151 (97.8%)	+	C.colli
RC105	412 (99.3%)	160 (99.4%)	481 (99.4%)	171 (100%)	126 (97.0%)	567 (99.0%)	302 (100%)	294 (98.3%)	821 (98.0%)	147 (99.3%)	464 (99.8%)	257 (100%)	1170 (99.6%)	+	C.colli
RG106	412 (99.3%)	160 (99.4%)	481 (99.4%)	171 (100%)	126 (97.0%)	567 (99.0%)	302 (100%)	294 (98.3%)	821 (98.0%)	147 (99.3%)	464 (99.8%)	257 (100%)	1170 (99.6%)	+	C.colli
RC116	311 (74.9%)	160 (99.4%)	481 (99.4%)	171 (100%)	126 (97.0%)	567 (99.0%)	302 (100%)	294 (98.3%)	821 (98.0%)	147 (99.3%)	464 (99.8%)	257 (100%)	1170 (99.6%)	+	C.colli
RC126	412 (99.3%)	160 (99.4%)	481 (99.4%)	171 (100%)	126 (97.0%)	567 (99.0%)	302 (100%)	294 (98.3%)	821 (98.0%)	147 (99.3%)	464 (99.8%)	257 (100%)	1170 (99.6%)	+	C.colli
RC127	412 (99.3%)	160 (99.4%)	481 (99.4%)	171 (100%)	126 (97.0%)	567 (99.0%)	302 (100%)	294 (98.3%)	820 (97.9%)	147 (99.3%)	464 (99.8%)	257 (100%)	1170 (99.6%)	+	C.colli
RC148	389 (93.7%)	160 (99.4%)	481 (99.4%)	171 (100%)	126 (97.0%)	567 (99.0%)	302 (100%)	294 (98.3%)	820 (97.9%)	147 (99.3%)	465 (100%)	254(98.8%)	1170 (99.6%)	+	C.colli
RC182	412 (99.3%)	160 (99.4%)	481 (99.4%)	171 (100%)	126 (97.0%)	567 (99.0%)	302 (100%)	294 (98.3%)	820 (97.9%)	147 (99.3%)	432 (92.9%)	257 (100%)	1170 (99.6%)	+	C.colli
RC264	412 (99.3%)	160 (99.4%)	481 (99.4%)	171 (100%)	126 (97.0%)	567 (99.0%)	302 (100%)	294 (98.3%)	820 (97.9%)	147 (99.3%)	465 (100%)	257 (100%)	1170 (99.6%)	+	C.colli
RC269	412 (99.3%)	160 (99.4%)	481 (99.4%)	171 (100%)	126 (97.0%)	567 (99.0%)	302 (100%)	294 (98.3%)	820 (97.9%)	147 (99.3%)	464 (99.8%)	257 (100%)	1170 (99.6%)	+	C.colli
RC281	412 (99.3%)	160 (99.4%)	481 (99.4%)	171 (100%)	126 (97.0%)	567 (99.0%)	302 (100%)	294 (98.3%)	820 (97.9%)	147 (99.3%)	464 (99.8%)	257 (100%)	1170 (99.6%)	+	C.colli
RC282	412 (99.3%)	160 (99.4%)	481 (99.4%)	171 (100%)	126 (97.0%)	567 (99.0%)	302 (100%)	294 (98.3%)	820 (97.9%)	147 (99.3%)	464 (99.8%)	257 (100%)	1170 (99.6%)	+	C.colli
RC284	412 (99.3%)	160 (99.4%)	481 (99.4%)	171 (100%)	126 (97.0%)	567 (99.0%)	302 (100%)	294 (98.3%)	820 (97.9%)	147 (99.3%)	464 (99.8%)	257 (100%)	1170 (99.6%)	+	C.colli
RC285	412 (99.3%)	160 (99.4%)	481 (99.4%)	171 (100%)	126 (97.0%)	567 (99.0%)	302 (100%)	294 (98.3%)	820 (97.9%)	147 (99.3%)	464 (99.8%)	257 (100%)	1170 (99.6%)	+	C.colli
RC289	412 (99.3%)	160 (99.4%)	481 (99.4%)	171 (100%)	126 (97.0%)	567 (99.0%)	302 (100%)	294 (98.3%)	820 (97.9%)	147 (99.3%)	464 (99.8%)	257 (100%)	1168(99.4%)	+	C.colli
RC291														+	C.colli
RC382	412 (99.3%)	160 (99.4%)	481 (99.4%)	171 (100%)	126 (97.0%)	567 (99.0%)	302 (100%)	294 (98.3%)	820 (97.9%)	147 (99.3%)	464 (99.8%)	257 (100%)	1170 (99.6%)	+	C.colli
RC383	412 (99.3%)	160 (99.4%)	481 (99.4%)	171 (100%)	126 (97.0%)	567 (99.0%)	302 (100%)	294 (98.3%)	820 (97.9%)	147 (99.3%)	464 (99.8%)	257 (100%)	1170 (99.6%)	+	C.colli
RC386	412 (99.3%)	160 (99.4%)	481 (99.4%)	171 (100%)	126 (97.0%)	567 (99.0%)	302 (100%)	294 (98.3%)	820 (97.9%)	147 (99.3%)	464 (99.8%)	257 (100%)	1170 (99.6%)	+	C.colli
RC387	412 (99.3%)	160 (99.4%)	481 (99.4%)	171 (100%)	126 (97.0%)	567 (99.0%)	302 (100%)	294 (98.3%)	820 (97.9%)	147 (99.3%)	464 (99.8%)	257 (100%)	1170 (99.6%)	+	C.colli
RC430	412 (99.3%)	160 (99.4%)	481 (99.4%)	171 (100%)	126 (97.0%)	567 (99.0%)	302 (100%)	294 (98.3%)	820 (97.9%)	147 (99.3%)	459 (98.7%)	226 (87.9%)	1170 (99.6%)	+	C.colli
RC415	412 (99.3%)	160 (99.4%)	481 (99.4%)	171 (100%)	126 (97.0%)	567 (99.0%)	302 (100%)	294 (98.3%)	820 (97.9%)	147 (99.3%)	464 (99.8%)	257 (100%)	1170 (99.6%)	+	C.colli
RC428	412 (99.3%)	160 (99.4%)	481 (99.4%)	171 (100%)	126 (97.0%)	567 (99.0%)	302 (100%)	294 (98.3%)	820 (97.9%)	147 (99.3%)	464 (99.8%)	257 (100%)	1170 (99.6%)	+	C.colli
RC459					126 (97.0%)	567 (99.0%)	146 (48.3%)	295(98.7%)	599 (71.5%)				1170 (99.6%)	+	C.colli
12502	413 (99.5%)	160 (99.4%)	481 (99.4%)	171 (100%)	127 (97.7%)	569 (99.3%)	301 (99.7%)	294 (98.3%)	825 (98.4%)	147 (99.3%)	464 (99.8%)	254 (98.8%)	1171 (99.7%)	+	C.jejuni
RC018			344 (71.1%)		127 (97.7%)	569 (99.3%)	302 (100%)		821 (98.0%)				332 (28.3%)	+	C.colli
RC039	412 (99.3%)	160 (99.4%)	481 (99.4%)	169 (98.8%)	126 (97.0%)	541 (94.4%)	302 (100%)	294 (98.3%)	821 (98.0%)	147 (99.3%)	464 (99.8%)	257 (100%)	1170 (99.6%)	+	C.jejuni
RC104	340 (81.9%)	155 (96.3%)	477 (99.4%)	156 (91.2%)	115 (88.5%)	567 (99.0%)	272 (90.1%)	296 (99.0%)	782 (93.3%)	146(98.6%)	465 (100%)	255 (99.2%)	1158 (98.6%)	+	C.jejuni
RC168	412 (99.3%)	160 (99.4%)	481 (99.4%)	171 (100%)	126 (97.0%)	526 (91.8%)	302 (100%)	294 (98.3%)	820 (97.9%)	147 (99.3%)	464 (99.8%)	257 (100%)	1170 (99.6%)	+	C.jejuni
RC169	412 (99.3%)	160 (99.4%)	481 (99.4%)	171 (100%)	126 (97.0%)	567 (99.0%)	302 (100%)	294 (98.3%)	820 (97.9%)	147 (99.3%)	464 (99.8%)	257 (100%)	1170 (99.6%)	+	C.jejuni
RC179	412 (99.3%)	160 (99.4%)	481 (99.4%)	171 (100%)	126 (97.0%)	567 (99.0%)	302 (100%)	294 (98.3%)	820 (97.9%)	147 (99.3%)	464 (99.8%)	257 (100%)	1170 (99.6%)	+	C.jejuni
RC186	412 (99.3%)	160 (99.4%)	481 (99.4%)	171 (100%)	126 (97.0%)	567 (99.0%)	302 (100%)	294 (98.3%)	820 (97.9%)	147 (99.3%)	464 (99.8%)	257 (100%)	1170 (99.6%)	+	C.jejuni
RC185	412 (99.3%)	160 (99.4%)	481 (99.4%)	171 (100%)	126 (97.0%)	567 (99.0%)	302 (100%)	294 (98.3%)	820 (97.9%)	147 (99.3%)	464 (99.8%)	257 (100%)	1170 (99.6%)	+	C.jejuni
RC220	412 (99.3%)	160 (99.4%)	481 (99.4%)	171 (100%)	126 (97.0%)	567 (99.0%)	302 (100%)	294 (98.3%)	820 (97.9%)	147 (99.3%)	416 (89.5%)	257 (100%)	1170 (99.6%)	+	C.jejuni
RC270	412 (99.3%)	160 (99.4%)	481 (99.4%)	171 (100%)	126 (97.0%)	567 (99.0%)	302 (100%)	294 (98.3%)	820 (97.9%)	147 (99.3%)	464 (99.8%)	257 (100%)	1170 (99.6%)	+	C.jejuni
RC280	367 (88.4%)	156 (96.9%)	481 (99.4%)	171 (100%)	126 (97.0%)	567 (99.0%)	302 (100%)	294 (98.3%)	820 (97.9%)	147 (99.3%)	464 (99.8%)	226 (87.9%)	1170 (99.6%)	+	C.jejuni
RC317	412 (99.3%)	160 (99.4%)	481 (99.4%)	171 (100%)	126 (97.0%)	567 (99.0%)	302 (100%)	294 (98.3%)	820 (97.9%)	147 (99.3%)	464 (99.8%)	257 (100%)	1170 (99.6%)	+	C.jejuni
RC410														+	C.jejuni
RC427	412 (99.3%)	160 (99.4%)	481 (99.4%)	171 (100%)	126 (97.0%)	567 (99.0%)	302 (100%)	294 (98.3%)	820 (97.9%)	147 (99.3%)	449 (96.6%)	257 (100%)	1170 (99.6%)	+	C.jejuni
RC508	412 (99.3%)	160 (99.4%)	481 (99.4%)	171 (100%)	126 (97.0%)	567 (99.0%)	302 (100%)	294 (98.3%)	820 (97.9%)	147 (99.3%)	464 (99.8%)	257 (100%)	1170 (99.6%)	+	C.jejuni
RC517	412 (99.3%)	160 (99.4%)	481 (99.4%)	171 (100%)	126 (97.0%)	567 (99.0%)	302 (100%)		820 (97.9%)	147 (99.3%)	464 (99.8%)	257 (100%)		+	C.jejuni
RC526			422 (87.2%)		126 (97.0%)			147 (49.2%)	493 (58.8%)					+	C.jejuni

### Relative motility of isolates

**Figure 2:**
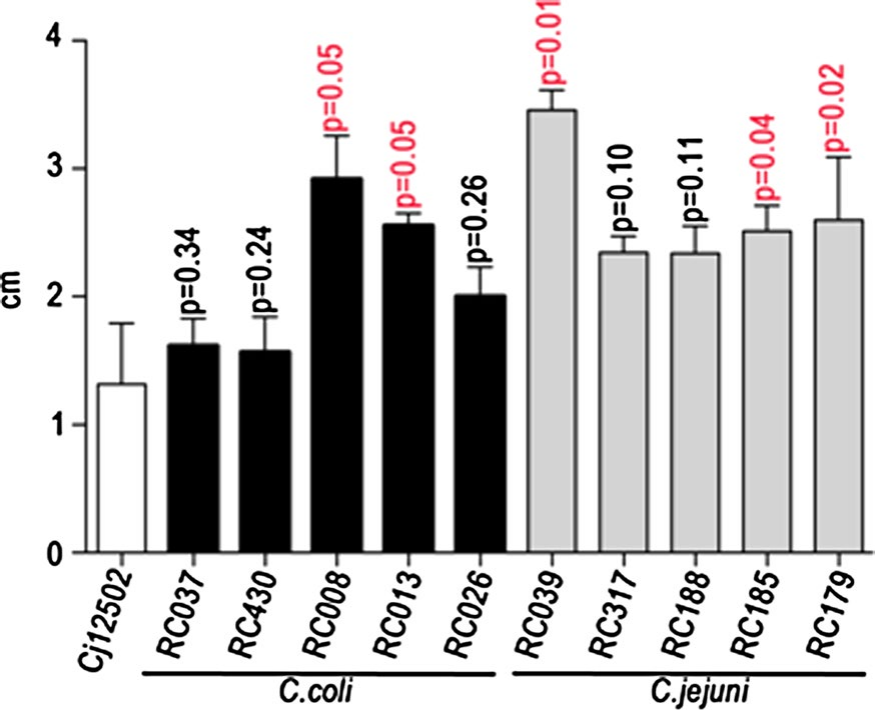
Relative motility assay of *hcp* ^+^ *C. jejuni* and *C. coli* isolates. The motility of five *hcp* positive *C. jejuni* and *C. coli* isolates was compared to *hcp* ^+^ control strain, *C. jejuni* 12502 in 0.4% BHI agar. Results are the mean of three separate experiments. The statistical significance was determined using the Student *t* test.

Motility represents and important virulence factor in campylobacters increasing their ability to invade and colonise the gut epithelium. We have next investigated the motility of the *hcp* positive *C. jejuni* and *C. coli* isolates in order to differentiate and select them for further pathogenicity investigations, using motility as an exclusion factor. Our initial results show that none of the *hcp*^+^*C. jejuni* and *C. coli* isolates displayed motility significantly less than that of the control strain *C. jejuni* 12502 (data not shown), p < 0.05 and five isolates from each species were selected for further study (Figure 2), based on their high levels of motility.

### AFLP analysis of a subset of *C. jejuni* and *C. coli* strains

**Figure 3:**
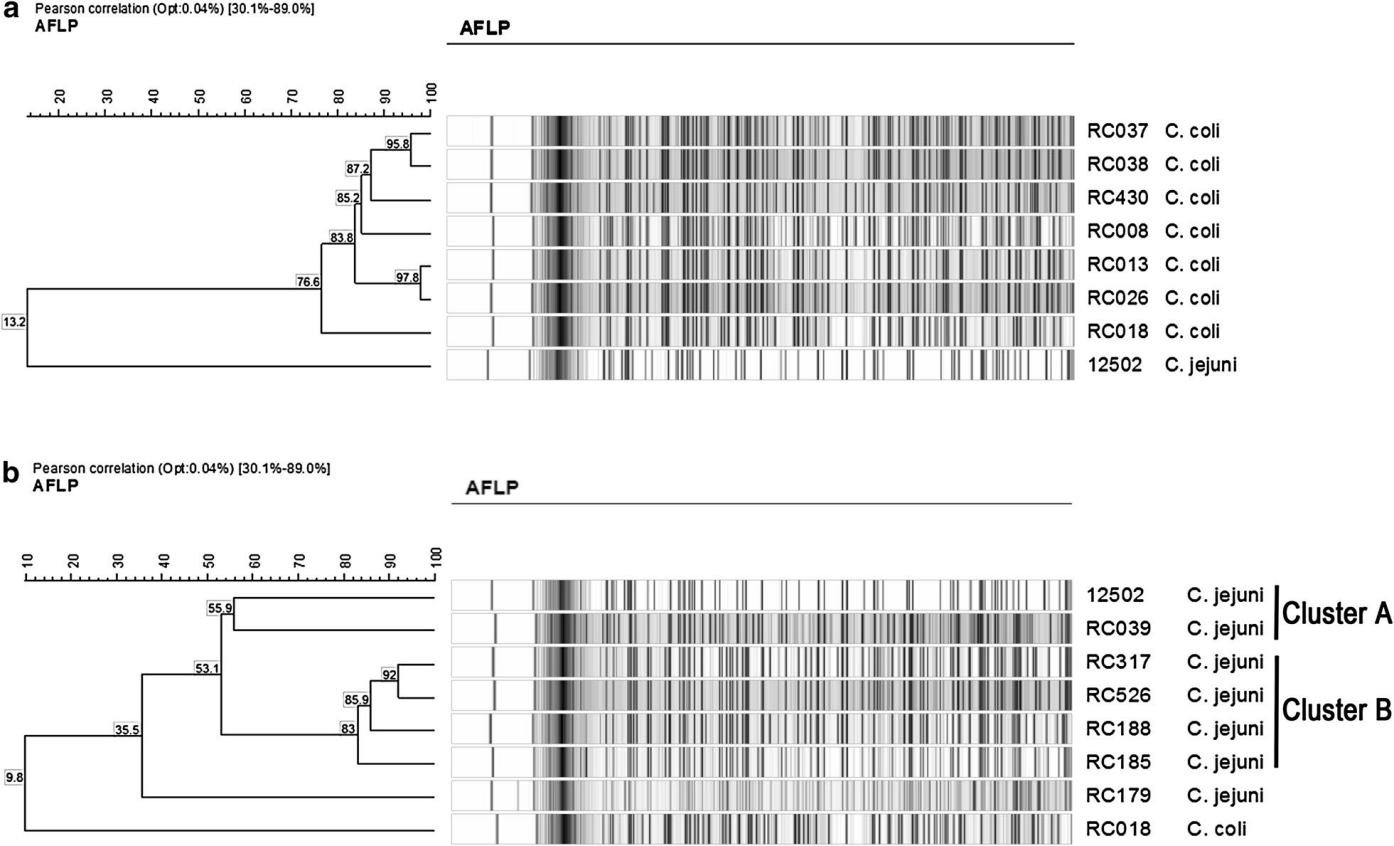
AFLP analysis of *C. coli* and *C. jejuni* *hcp* positive chicken isolates. **a** shows the AFLP fingerprints of the five most motile *C. coli* isolates and **b** the *C. jejuni* isolates. The percentage of amino acid identity between banding patterns is indicated. For comparison we have used the *hcp* positive *C. jejuni* 12502 and the negative isolate RC018.

AFLP was used to determine the genetic difference between the five strains of *C. coli* and of *C. jejuni* selected for further study. The *C. coli* isolates; RC037, RC038 and RC013, 026 produced two clusters with 95.8% genetic similarity and 97.8% respectively. The degree of genetic similarity between these two clusters and *C. coli* isolates RC430 and RC008 was over 80%. When the *hcp*^+^ isolates were compared with the negative control, RC018, the degree of similarity was also very high, >70%, (Figure 3a). The *C. jejuni* 12502 control has been included in the AFLP diagram to specifically emphasize the genetic differences between *C. jejuni* and C. coli isolates and similarly for *C. coli* RC018 for the C. jejuni clustering.

*C. jejuni* isolates showing higher motility were also investigated by AFLP (panel b). Isolate RC039 formed a separate cluster with the control strain *C. jejuni* 12502 at a degree of similarity of over 50% (cluster A) and isolates RC317, RC526, RC188 and RC185 clustered together at a similarity >80% (cluster B). When cluster A was compared to cluster B the degree of similarity was approximately 50%. Isolate RC179 showed a low degree of similarity, approximately 35%, when compared to cluster A and B.

### *In vitro* cell invasion abilities of *hcp*^+^ isolates

**Figure 4:**
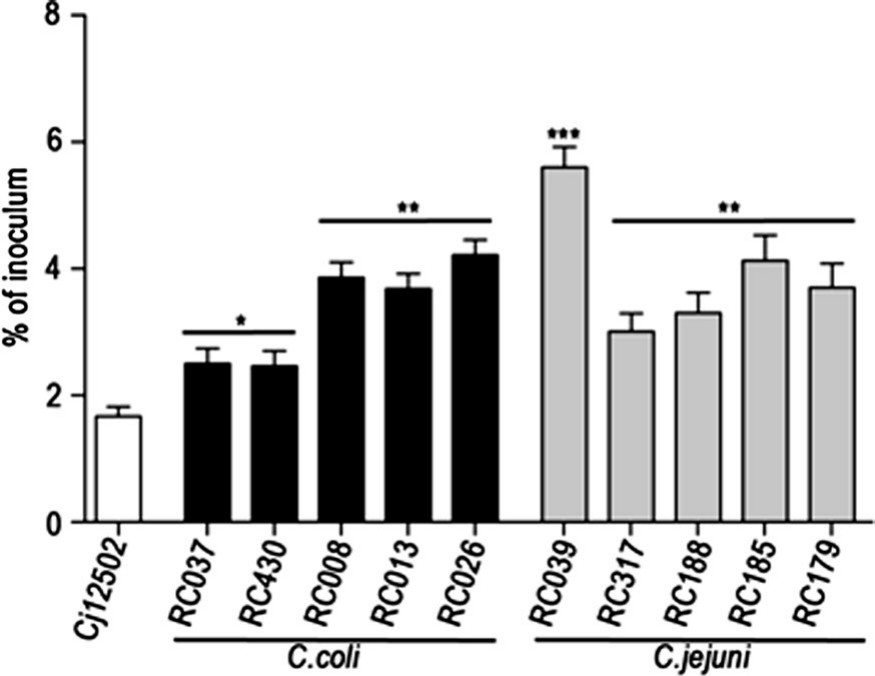
Invasive ability of *C. coli* and *C. jejuni* isolates. Invasion of HCT-8 cells of *C. coli* (RC037, 430, 008, 013 and 026) and *C. jejuni* (RC039, 317, 188, 185 and 179) isolates. Statistical significance (Student *t* test) relative to the level of *C. jejuni* 12502 is indicated. The experiments were done in triplicate and on three separate occasions. The *error bars* represent standard deviations.

The ability of *hcp*^+^*C. coli* and *C. jejuni* isolates to invade HCT-8 cells was studied using *C. jejuni* 12502 as a reference strain (Figure 4). Using gentamicin, higher invasiveness rates were determined for *C. coli* isolates RC037 and RC130 when compared to *C. jejuni* invasiveness (p < 0.05). For Approximately 4% of the inoculums was internalised with *C. coli* isolates RC008, RC013 and RC026, and *C. jejuni* isolates RC317, RC188, RC185 and RC179: significantly more the level of less than 2% seen for the control strain (p < 0.01). *C. jejuni* isolate RC039 was most invasive, with approximately 6% of the inoculum being internalised (p < 0.001). A positive correlation between motility and invasiveness was seen for of all the *hcp*^+^*C. coli* and *C. jejuni* isolates. The *hcp*^+^ negative isolate (RC018) displayed invasiveness of less than 0.01% of the inoculum (data not shown).

### Capsule polysaccharide profiles of *C. coli* isolates

**Figure 5:**
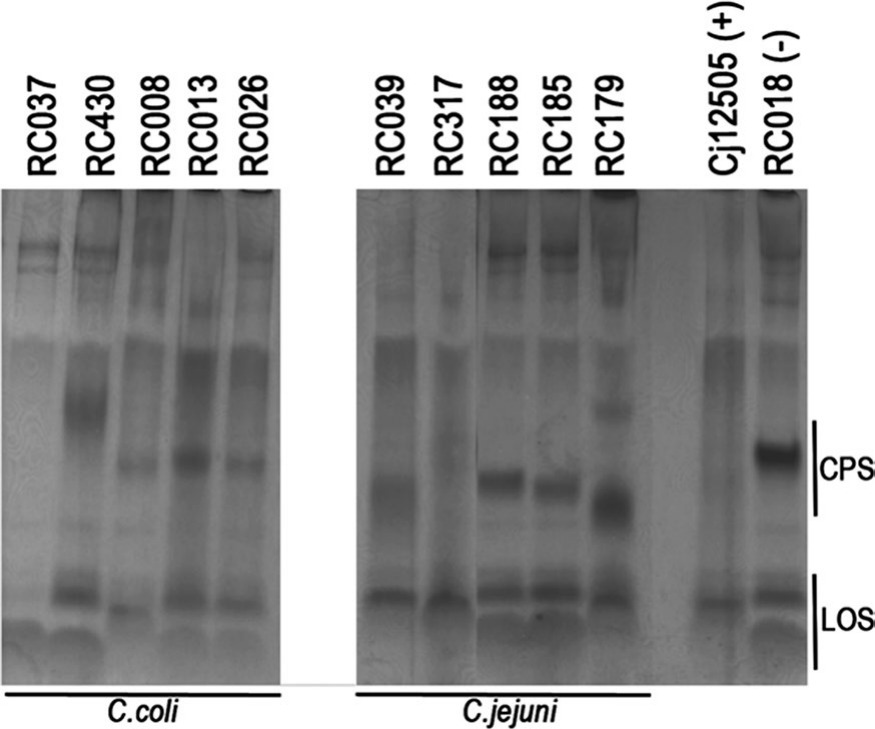
Capsule polysaccharides profiles of *hcp* positive isolates. Capsular polysaccharides were separated in 10% SDS-PAGE gels and stained with Alcian blue. The profiles of *C. jejuni* 12502 and *C. coli* RC018 served as controls for lipo-oligosaccharide (LOS).

Five isolates were chosen for study based on the high levels recorded in the motility determinations noted above. *C. coli* isolates RC037 and RC038 showed low amounts of CPS with most of the polysaccharides having very high molecular weights, similar to the control strain *C. jejuni* 12502. Isolates RC008, RC013 and RC026 had CPS molecular weights similar to the negative control, isolate RC018. The *C. jejuni* positive isolates had very different CPS profiles, with only the isolates RC526 and RC018 having profiles similar to each other, but dissimilar to *C. jejuni* 12502. Lower amounts of LOS were also detected, following staining with Alcian blue, for isolates RC039, RC526 and RC179, Figure 5. Due to the diversity of the capsule profiles obtained, between the different isolates, a capsule deficient mutant has not been used for comparison. We will focus in the future on the most virulent isolate and a mutant will be created for control purposes.

### Bile salts resistance

The ability of hcp^+^ chicken isolates to survive in the presence of bile salts (Table 3). Most of the *C. coli* isolates showed no resistance to bile salts (RC037, RC430, RC013 and RC026) with only isolate RC008 displaying intermediate resistance (i.e. lower counts compared to the control strain *C. jejuni* 12502). A similar pattern was also found with *C. jejuni*, with isolates RC039, RC317, RC188 and RC185 showing no resistance to bile salts in vitro.

**Table 3:** Resistance to bile salts

Isolate	Control	*Campylobacter coli*					*Campylobacter jejuni*				
	12502	RC037	RC430	RC008	RC013	RC026	RC039	RC317	RC188	RC185	RC179
Bile salt resistance	+	–	–	±	–	–	–	–	–	–	±

(+) resistance, (±) intermediate, (−) sensitive.

## Discussion

Campylobacters are the main cause of in human food poisoning in Europe and North America, with chicken meat identified as a major source of campylobacters, which grow profusely in the gut and crop of broiler chickens. The European Food Safety Authority (EFSA) stated that reducing the numbers of *Campylobacter* in the intestines at slaughter 1,000-fold would reduce the public health risk by at least 90%. Previous studies have shown that the virulence abilities, of *Campylobacter* chicken isolates, are increased compared to human isolates [13, 37]. The Type VI secretion system novel protein translocation system has been described as a potentially new virulence factor active in vitro [11] and in vivo [14]. However, the role of T6SS in acute human disease remains to be determined, as the biological mechanisms involved are not yet fully understood. This study investigated the prevalence of T6SS in *C. coli* and *C. jejuni* chicken isolates and to characterise the virulence potential of these isolates in vitro.

The *hcp* gene is currently used as the main indicator for the presence of T6SS *C. jejuni* [11]. The incidence of *hcp* has been reported as being relatively higher in *C. jejuni* isolates from chickens in Asia, whilst and only a small proportion of chicken isolates in the UK were identified as positive for T6SS [13]. Given the pathogenic potential of *hcp*^+^ positive campylobacters, this study investigated, by multiplex PCR, the presence of the *hcp* gene in both *C. coli* and *C. jejuni* isolates obtained from retail chicken produced in Northern Ireland. It has been reported that the prevalence of *C. coli* isolates in retail chicken is much lower than that of *C. jejuni* [18] but this study found that the incidence of *hcp* was higher in *C. coli, than in C. jejuni,* hence these strains could potentially display increased virulence abilities. However, epidemiological studies have found that only 7% of reported cases of campylobacteriosis are caused by *C. coli* and 93% by *C. jejuni*, with most of the *C. coli* cases being recorded in older patients [38]. No epidemiological connection to the presence of *hcp* has been reported in *C. coli.* Our results show that the majority of the *C. coli* and *C. jejuni* isolates contained the complete set of ORFs required for a fully active T6SS. The characterization of the T6SS ORF was undertaken as it has been suggested [13] that the detection of the *hcp* gene alone might not actually indicate the presence of a full T6SS locus. This was demonstrated in Spanish isolates where it was shown that only 14% of the strains (n = 9) had a complete T6SS ORF [19].

In this study, analysis of the genetic similarities between isolates revealed similar results to those previously reported, i.e. suggesting that *C. coli* isolates were less diverse, based on the AFLP profiles, that *C. jejuni* isolates [39], probably due to *C. coli* showing more host specificity than *C. jejuni* [40].

*Campylobacter* species have emerged as gastrointestinal pathogens in humans and commensals in birds [41] with motility as a phenotypic feature required to achieve both colonization [42] and internalization in surface intestinal epithelial layer [43]. The results reported above show that all of the *hcp*^+^ chicken isolates had greater motility than an *hcp*^+^ human isolate (*C. jejuni* 12502), but no direct link between the presence of the *hcp* gene and motility in Campylobacter has yet been reported. However, the observations reported above are supported by studies with other Gram-negative bacteria such as *Aeromonas hydrophila*. *A. hydrophila* produces a cytotoxic enterotoxin, and associated with this toxin is a type II secretion system. However, it has also been reported that *A. hydrophila* possess a T6SS and that *hcp* increases not only motility but also protease and biofilm formation, thus significantly influencing the pathogenicity and survival of this microorganism [44].

In *Campylobacter*, motility is recognised as an important virulence factor [1] and the chicken isolates in this study were shown, not only to be highly motile, but also very invasive when in vitro gentamicin protection assays were performed with human intestinal cells [33]. In general *hcp*^−^ strains were poorly invasive, e.g. *C. jejuni* 11168, with less than 0.1% of the inoculum invading the epithelial cells [34] but with *hcp*^+^ strains over 1% invades [14]. All of the *hcp*^+^ chicken isolates investigated in this study demonstrated greater invasiveness in the gentamicin protection assay [14], when compared to the control. The involvement of *hcp* in *C. coli* in vitro virulence is still under debate since our genomic sequencing results show that even the isolates with an incomplete ORF are not significantly different in virulence compared to strains with complete ORFs. This suggests that in these isolates other virulence factors might also be involved.

To act as a haemolysin, *hcp* requires to be synthesised and subsequently injected into the host cell, using the fully functional T6SS. This process will require modifications of the bacterial surface structures, especially its polysaccharide capsule, to allow the T6SS to act effectively. The decrease in capsule production has been reported as necessary for effective T6SS mediated toxicity and virulence [11]. Accordingly, the capsule polysaccharides of *hcp*^+^ isolates were studied to detect any differences in the molecular weights, or amounts, between these isolates. With isolate RC039, less CPS appeared to coincide with greater virulence characteristics, however this was not the case with the other isolates. However, in *Campylobacter* de-capsulation can occur in the presence of epithelial cells [34], hence the CPS profiles may not reflect their state during pathogenesis.

In order to successfully colonise humans, *Campylobacter* will have to be resistant to the potentially lethal conditions present in the intestinal tract, and possess resistance to bile salts [45]. It has been suggested that deoxycholic acid, at physiological levels found in the human gut, can inhibit cell growth in *C. jejuni*, however when the concentration falls to those found in the proximal colon [46], the T6SS system is expressed and this contributes to colonic inflammation in humans [14]. In this study the resistance of the isolates to bile salts were compared with the human isolate *C. jejuni* 12502, revealing that most isolates had no resistance to levels of bile salts previously described as minimally inhibitory concentrations [45].

## Conclusion

Taken together, all these results show that the *hcp* gene and the complete T6SS are prevalent in *C. coli* as well as in *C. jejuni* isolates obtained from retail chicken at a very high rate. Overall the results suggest that *C. jejuni* and *C. coli* chicken isolates containing the complete T6SS ORFs potentially have greater virulence in vitro, than do reference strains, but the consequences of these factors, in vivo, has yet to be determined.

### Availability of supporting data

The data set supporting the results of this article are available in the European Nucleotide Archive under the project accession number PRJEB9722.
